# e-Cigarette Vapour Condensate Reduces Viability and Impairs Function of Human Osteoblasts, in Part, via a Nicotine Dependent Mechanism

**DOI:** 10.3390/toxics10090506

**Published:** 2022-08-28

**Authors:** Thomas Nicholson, Lauren Davis, Edward T. Davis, Matthew Newton Ede, Aaron Scott, Simon W. Jones

**Affiliations:** 1 Institute of Inflammation and Ageing, MRC-ARUK Centre for Musculoskeletal Ageing Research, Institute of Inflammation and Ageing, University of Birmingham, Birmingham B15 2TT, UK; 2 Birmingham Acute Care Research Group, Institute of Inflammation and Ageing, University of Birmingham, Birmingham B15 2TT, UK; 3 Royal Orthopaedic Hospital, Bristol Road South, Birmingham B15 2TT, UK

**Keywords:** electronic cigarettes, osteoblast, e-cigarette, vaping, viability, bone, osteoprotegerin, human primary cells

## Abstract

Cigarette consumption negatively impacts bone quality and is a risk-factor for the development of multiple bone associated disorders, due to the highly vascularised structure of bone being exposed to systemic factors. However, the impact on bone to electronic cigarette (e-cigarette) use, which contains high doses of nicotine and other compounds including flavouring chemicals, metal particulates and carbonyls, is poorly understood. Here, we present the first evidence demonstrating the impact of e-cigarette vapour condensate (replicating changes in e-cigarette liquid chemical structure that occur upon device usage), on human primary osteoblast viability and function. 24 h exposure of osteoblasts to e-cigarette vapour condensate, generated from either second or third generation devices, significantly reduced osteoblast viability in a dose dependent manner, with condensate generated from the more powerful third generation device having greater toxicity. This effect was mediated in-part by nicotine, since exposure to nicotine-free condensate of an equal concentration had a less toxic effect. The detrimental effect of e-cigarette vapour condensate on osteoblast viability was rescued by co-treatment with the antioxidant N-Acetyl-L-cysteine (NAC), indicating toxicity may also be driven by reactive species generated upon device usage. Finally, non-toxic doses of either second or third generation condensate significantly blunted osteoblast osteoprotegerin secretion after 24 h, which was sustained for up to 7 days. In summary we demonstrate that e-cigarette vapour condensate, generated from commonly used second and third generation devices, can significantly reduce osteoblast viability and impair osteoblast function, at physiologically relevant doses. These data highlight the need for further investigation to inform users of the potential risks of e-cigarette use on bone health, including, accelerating bone associated disease progression, impacting skeletal development in younger users and to advise patients following orthopaedic surgery, dental surgery, or injury to maximise bone healing.

## 1. Introduction

Multiple meta-analyses have reported that a history of cigarette smoking is associated with significantly reduced bone mineral density (BMD), increased risk of fracture and reduced fracture healing, in comparison to age, sex and BMI-matched non-smokers [1]. It is also apparent that such smoking-associated effects are cumulative, demonstrating a positive correlation with pack year history [2,3,4]. Furthermore, fracture risk in smoking cohorts is greater than in non-smokers when corrected for BMD, indicating that smoking may directly impact bone architecture and quality. Indeed, a decrease in trabecular bone mass and increased trabecular separation has been reported in older smokers [5], while in younger individuals smoking is associated with a reduction in trabecular bone volume, independent of age, BMI, activity level and calcium intake [6]. Recent studies have also demonstrated that smoking is independently associated with increased post-surgery complications such as infection and aseptic loosening following arthroplasty [7,8,9,10].

While cigarette consumption has declined over the past decade, the use of electronic cigarettes (e-cigarettes) or vaping has risen dramatically, partly due to being regarded as a safer alternative to smoking, although, 8% of current EC users in the UK have never smoked a cigarette [11,12,13]. Increased use of e-cigarettes will undoubtedly make a significant contribution towards harm reduction in comparison to cigarettes. However, e-cigarette usage still results in systemic exposure to numerous and potentially harmful vapour constituents, particularly to highly vascularised tissues such as the bone. In support of this, Agoons et al. recently reported that e-cigarette users have a 46% higher prevalence of fractures, in comparison to those who have never used e-cigarettes based on a cohort of 4519 individuals [14].

E-cigarette vapour is much less complex than cigarette smoke, yet many harmful constituents of cigarette smoke are found in e-cigarette vapour. Upon thermal decomposition, e-liquid humectants propylene glycol (PG) and vegetable glycerine (VG) form products such as acrolein and formaldehyde, commonly termed reactive carbonyl species (RCS), which are causatively linked to systemic harm [15,16]. Furthermore, since their invention in 2003, e-cigarette device technology has developed rapidly with current 3rd generation devices capable of delivering vapour at a much higher temperature than earlier models due to larger battery sizes. Consequently, this enables greater delivery of nicotine [17,18,19], increasing user satisfaction but also delivering much greater amounts of harmful RCS [20,21,22].

As to be expected, the majority of research on e-cigarettes to date has been carried out in models relevant to the lungs. Importantly, we and others have investigated the effect of vaping constituents on lung immune cells, reporting cytotoxic, proinflammatory and anti-phagocytic effects in alveolar macrophages [15,23]. Similar reductions in neutrophil function have also been reported, including reduced neutrophil migration and phagocytosis, suppression of NETosis and increased ROS production [24]. Additionally, dysfunctional cilia beat frequency and motility has been reported in human airway epithelial cells and normal human bronchial epithelial (NHBE) cells following cigarette vapour exposure [25,26,27,28].

However, there has been limited investigation into the impact of e-cigarette usage on bone physiology, particularly following long-term use [29,30]. There are also limited in vitro data, particularly utilising human osteoblasts. Typically exhibiting a large, cuboidal morphology, osteoblasts are the primary cell type responsible for bone formation, through secretion of collagenous and non-collagenous proteins and proteoglycans that in turn become mineralised.

Utilising a novel system previously described by our group [23], we have performed the first investigation into the effect of e-cigarette vapour condensate on human primary osteoblast viability and function. Importantly, we report the comparative effects of vaping constituents generated by 2nd generation and 3rd generation devices, which together account for 77% of devices used in the UK [31]. Finally, we have utilised both nicotine containing and nicotine-free vapour condensate, in addition to the antioxidant, N-acetyl cysteine (NAC) to investigate the contribution of the vapour constituents nicotine and RCS, respectively, on osteoblast viability.

## 2. Materials and Methods

### 2.1. Ethical Approval and Subject Recruitment

Femoral heads were collected from hip osteoarthritis (OA) patients undergoing orthopaedic joint replacement surgery at the Royal Orthopaedic Hospital (Birmingham, UK). All patient participants were recruited on a volunteer basis, after being fully informed of the study requirements by the clinical research staff, and providing written consent (NRES 16/SS/0172).

### 2.2. Primary Human Osteoblast Cell Culture 

Trabecular bone chips (<100 mm^3^) were obtained from the OA patient femoral heads using a Friedman Rongeur, washed three times in phosphate-buffered saline (PBS) [Life Technologies Ltd., Renfrew, UK] and once with high-glucose Dulbecco’s Modified Eagle Medium (DMEM) to remove excess fat, blood, marrow, and connective tissue. Bone chips were then cut into small pieces (<5 mm^3^) and transferred to a 25 cm^2^ vented flask containing primary human osteoblast media (DMEM, 10% FBS, 100 Units/mL Penicillin Streptomycin, 2 mM L-Glutamine, 1% NEAA, 2 mM β-glycerophosphate disodium salt hydrate, 50 ug/mL L-Ascorbic Acid, 10 nM Dexamethasone). Bone chips were incubated at 37 °C and 5% CO_2_ for 5 days before the initial media change. Following 5 days, differentiation media was changed every 3 days, and bone chips were removed once primary osteoblast cell coverage reached approximately 50% confluency. Upon reaching confluency, cells were passaged into a 75 cm^2^ flask. For all experiments primary human osteoblasts were limited to passage 5.

### 2.3. e-Cigarette Devices

Two popular devices in the UK were chosen for condensate generation, a 2nd generation device and 3rd generation device from Kanger tech Ltd., (Shenzhen, China). The 2nd generation device was fitted with a standard 650 mAh battery with a fresh 1.8 Ohm atomiser for each preparation, generating 7.6 W. The 3rd generation device, the most powerful of the devices, was fitted with a 3000 mAh battery with a fresh 0.15 Ohm atomizer fitted for each preparation, generating 75 W. The same devices were used for each condensate preparation.

### 2.4. e-Cigarette Vapour Condensate Collection

e-cigarette vapour condensate (ECVC) or nicotine-free e-cigarette vapour condensate (nfECVC) was collected from 2nd and 3rd generation e-cigarette devices, as previously described by Scott et al. [23]. Prior to use, e-Cigarette devices were cleaned and prepared with either 36 mg/mL nicotine flavourless liquid (Durasmoke^®^ Unflavored e-Liquid (50% PG/50% VG Base), American e-liquid Store, (Wauwatosa, WI, USA) or nicotine-free flavourless liquid (Durasmoke^®^ Unflavored eLiquid (50% PG/50% VG Base), American e-liquid Store, (Wauwatosa, WI, USA). Next, six tracheal suction taps (Unomedical, UK) were arranged in sequence and sealed with parafilm. EC devices were attached to the open end of tap 1, while tap 6 was connected to a vacuum tap by plastic tubing. Taps 2–6 were sealed inside 30 mL universal tubes with parafilm, to provide insulation and prevent cracking upon cooling. Next insulated taps were suspended in a dry ice/methanol bath and allowed to cool, tap 1 was kept outside the bath for observation of vapour production. The optimum puff duration of 3 s (previously determined by Scott et al.) was performed every 30 s until EC liquid was exhausted. Taps were then allowed to warm to room temperature, before centrifugation (2755× *g*, 5 min) to collect condensate. Condensate was pooled into a single 1.5 mL Eppendorf and stored at −40 °C for a maximum of 24 h before use.

### 2.5. Osteoblast Challenge and Intervention 

Challenge with ECVC, nfECVC, PG, VG and N-Acetyl-Cysteine (Sigma-Aldrich, St. Louis, MO, USA) were diluted in osteoblast differentiation media to concentrations detailed in individual Figure legends. Incubation periods are described per experiment as appropriate. 

### 2.6. Primary Human Osteoblast Viability and Cellular Morphology

Osteoblast viability was determined using CellTiter 96^®^ AQueous One Solution Cell Proliferation Assay (CTA) (Promega, UK) following the manufacturers protocol. Following addition of CTA reagent, cells were incubated in the dark for 3 h at 37 °C and 5% CO_2_. Absorbance at 490 nm was then immediately measured using a Synergy HT (BioTek, Santa Clara, CA, USA) plate reader. Additionally, in order to assess cell morphology, osteoblasts were imaged at 20× magnification using an SP8 Lightning confocal microscope (Leica microsystems, UK).

### 2.7. Quantification of OPG and RANK-L Secretion from Primary Human Osteoblasts

OPG and RANK-L protein in primary human osteoblast supernatants were quantified in duplicate using commercially available ELISAs (Osteoprotegerin/TNFRSF11B (R&D Systems, Minneapolis, MN, USA), Human TRANCE/RANK L/TNFSF11 (R&D Systems, 29, Minneapolis, MN, USA) following the manufacturer’s instructions. Absorbance was measured at 450 nm and 570 nm on a Synergy HT (BioTek, USA) plate reader.

### 2.8. Statistical Analysis

Data analysis was carried out using GraphPad Prism v8 statistical package. For data sets with 2 variables, significance was determined by 2-way ANOVA followed by Tukey’s multiple comparisons test where appropriate. For data sets with one variable, data was analysed using a non-parametric Kruskal–Wallis test, followed by post hoc Dunn’s multiple comparison tests. Data is presented as mean ± S.E.M with a *p* value < 0.05 considered statistically significant.

## 3. Results

### 3.1. ECVC from either 2nd or 3rd Generation e-Cigarette Devices Reduces Human Osteoblast Viability and Alters Cellular Morphology

**Figure 1:**
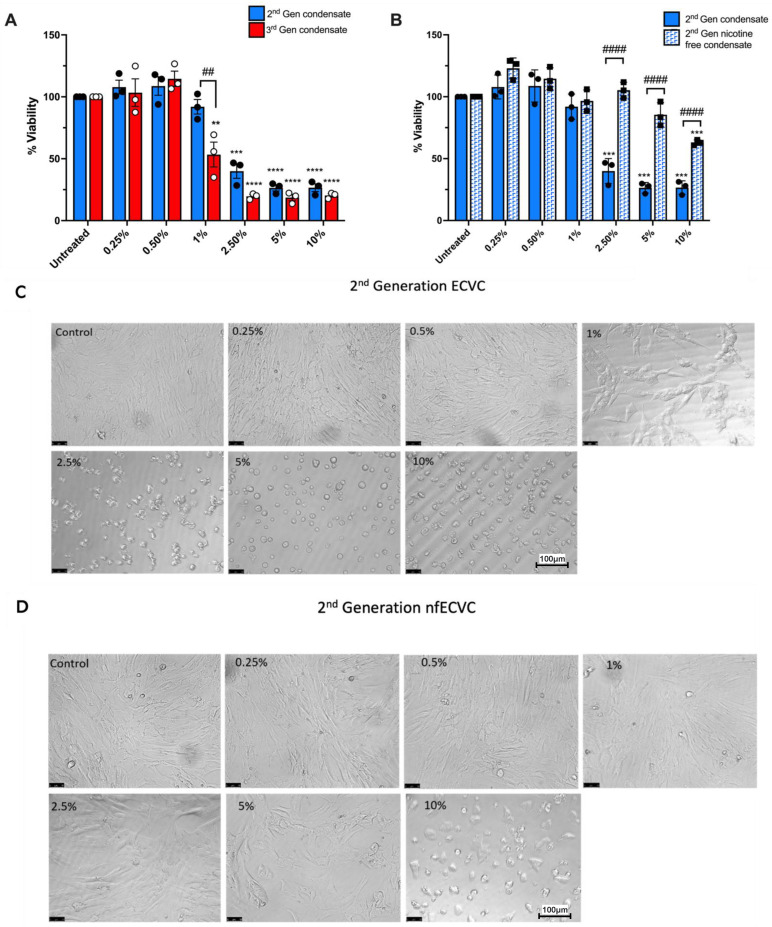

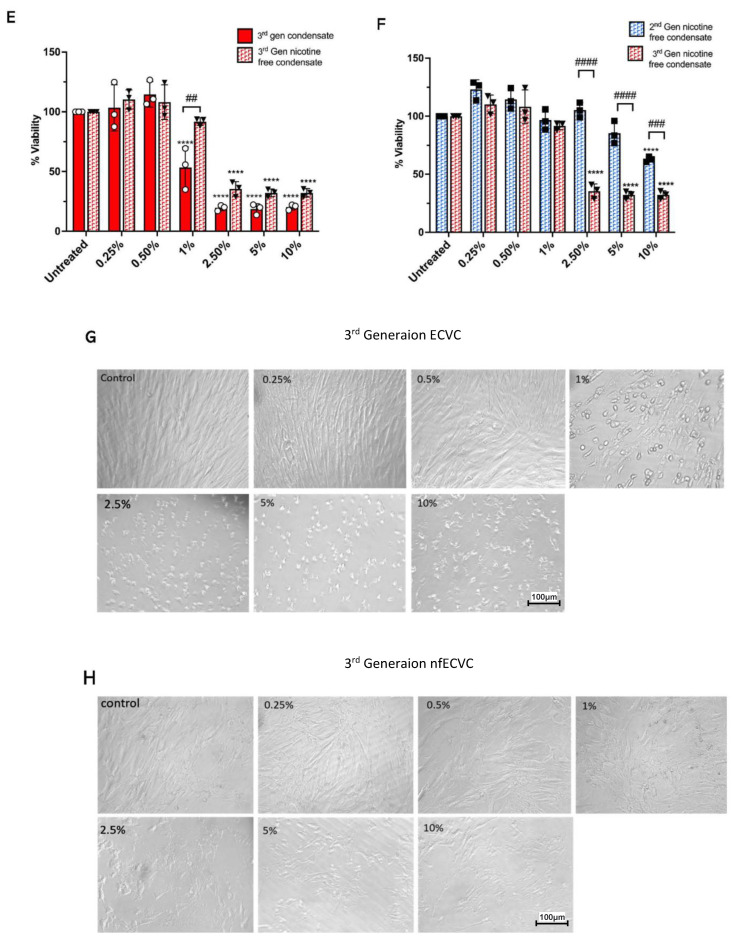
The effect of 2nd and 3rd generation ECVC and contribution of nicotine on human Primary osteoblast viability. (**A**) Osteoblast viability following 24 h exposure to 2nd or 3rd generation nicotine-containing ECVC (0.25–10%). (**B**) Osteoblast viability following 24 h exposure to either nicotine-containing or nicotine-free 2nd generation ECVC (0.25–10%). (**C**,**D**) Representative images of primary human osteoblasts treated with 2nd gen ECVC or nfECVF (0–10%). (**E**) Osteoblast viability following 24 h exposure to either nicotine-containing or nicotine-free 3rd generation ECVC (0.25–10%). (**F**) Osteoblast viability following 24 h exposure nicotine-free ECVC from either 2nd or 3rd generation devices (0.25–10%). (**G**,**H**) Representative images of primary human osteoblasts treated with 3rd generation ECVC or nfECVC (0–10%). Viability was inferred by 4 h incubation with cell titre aqueous assay. Images captured at 20× magnification. *n* = 3 patient replicates, with 5 biological replicates performed per patient. ** *p* < 0.01, *** *p* < 0.001, **** *p* < 0.0001 denoting a significant denoting a significant difference to relevant untreated control. ## *p* < 0.01, ### *p* < 0.001, #### *p* < 0.0001, denoting a significant difference between treatment groups.

Osteoblast viability was quantified and cellular morphology observed following 24 h exposure to increasing doses of ECVC (0.25% to 10%), generated from either 2nd or 3rd generation devices. ECVC at 0.25% or 0.5%, generated from either 2nd or 3rd generation devices, had no significant effect on osteoblast viability (Figure 1A) or cellular morphology (Figure 1C,G). However, in contrast to the 2nd generation device, 1% ECVC generated by the 3rd generation device resulted in a significant reduction in osteoblast viability (46.6% ± 10.0% *p* = 0.002), compared to untreated control cells (Figure 1A). At ECVC concentrations of 2.5% and greater, condensate generated by either 2nd or 3rd generation devices significantly reduced osteoblast viability (*p* < 0.0001, Figure 1A). Furthermore, osteoblasts exposed to 2nd or 3rd generation ECVC at concentrations of 2.5% or greater, showed clear signs of altered cellular morphology, with loss of spindle cell shape (Figure 1C,G).

### 3.2. ECVC from Nicotine-Free 3rd Generation Devices Has a Greater Effect on Reducing Osteoblast Viability than Nicotine-Free 2nd Generation e-Cigarette Devices 

To determine the extent that the observed reduction in osteoblast viability was attributable to nicotine content in the condensate, we compared the effects of nicotine-containing and nicotine-free ECVC (Figure 1B,E,F). As expected, from the 2nd generation devices, nicotine-containing ECVC at 2.5%, 5% and 10% induced a significant reduction in osteoblast viability (*p* > 0.0001) (Figure 1B,D). However, osteoblasts exposed to the nicotine-free 2nd generation condensate experienced a significantly lower loss in viability after; 10% nfECVC (37.2% ± 1.27% loss in viability), vs. just 2.5% ECVC *p* =< 0.0001) (Figure 1B,D). Exposure to 3rd generation condensate did not follow this pattern. 1% nfECVC challenge caused significantly less osteoblast toxicity than 1% nicotine containing ECVC (*p* = 0.001, Figure 1E,H). However, at higher concentrations, both nicotine-containing and nicotine-free condensate elicited a significant reduction in osteoblast viability, compared to untreated controls (Figure 1E,H). Whilst this effect was more pronounced with nicotine-containing condensate there was no significant difference between nicotine containing and nicotine free challenge at these doses (Figure 1E). Directly comparing nfECVC generated by 2nd generation and 3rd generation devices confirmed a greater toxic effect on osteoblasts when exposed to 3rd generation nfECVC at concentrations of 2.5% and above (*p* > 0.0001, Figure 1F).

### 3.3. Sub-Cytotoxic Doses of e-Cigarette Condensate Alters Human Osteoblast Function

**Figure 2:**
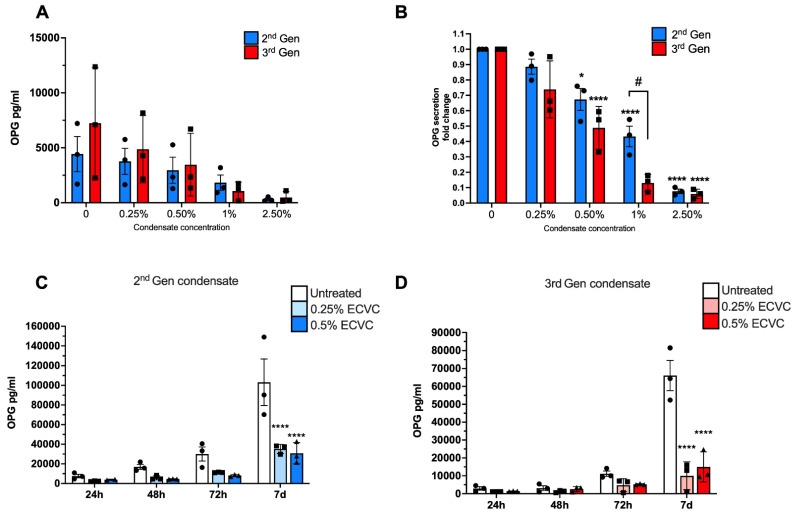

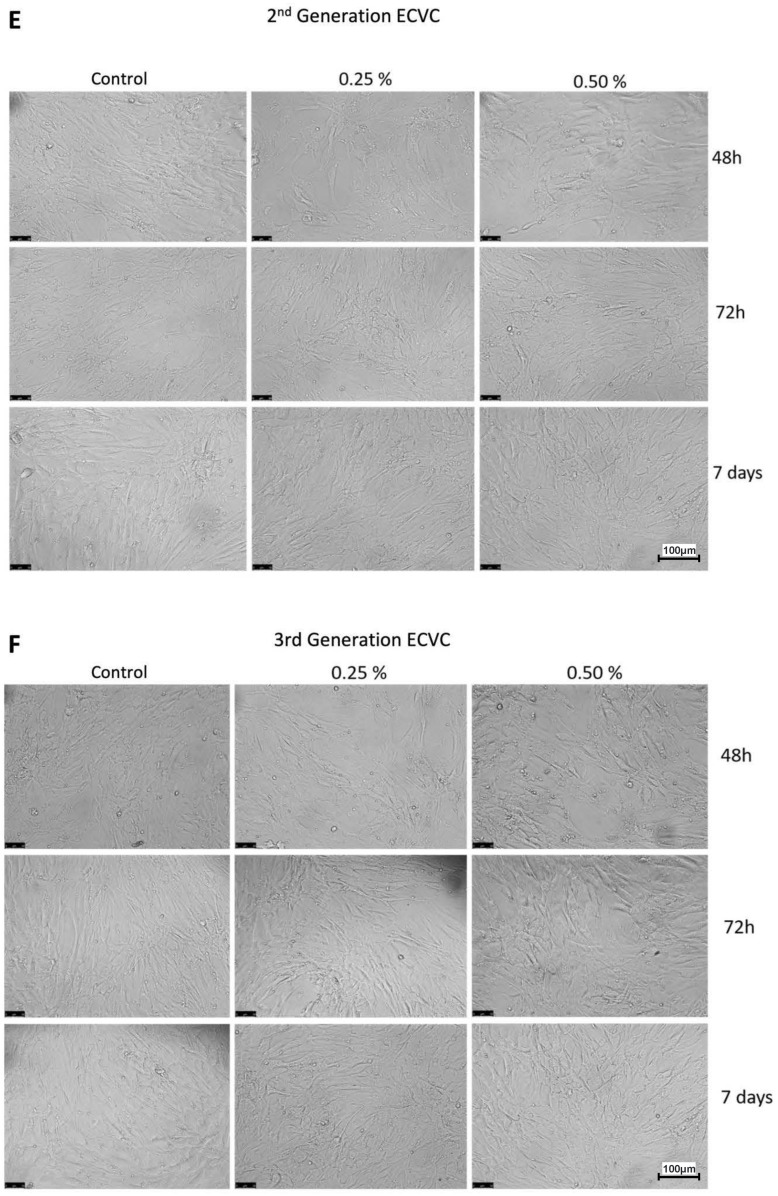
The impact of 2nd and 3rd generation ECVC on human primary osteoblast function. (**A**) Effect of 24 h exposure to 2nd and 3rd generation ECVC at concentrations from 0.25–2.5% on human primary osteoblast OPG secretion. (**B**) Effect of 24 h exposure to 2nd and 3rd generation ECVC at concentrations from 0.25–2.5% on human primary osteoblast OPG secretion expressed as fold change from untreated control. (**C**) Effect of 24–168 h (7d) exposure to 2nd generation ECVC at concentrations from 0.25–0.5% on human primary osteoblast OPG secretion. (**D**) Effect of 24–168 h (7d) exposure to 3rd generation ECVC at concentrations from 0.25–0.5% on human primary osteoblast OPG secretion. (**E**,**F**) Representative images of primary human osteoblasts treated with 0–0.5% 2nd and 3rd generation ECVC for up to 7 days. *n* = 3 patient replicates, with 3 biological replicates performed per patient. * *p* < 0.05, **** *p* < 0.0001 denoting a significant difference to relevant untreated control. # *p* < 0.05, denoting a significant difference between treatment groups.

Next, we investigated the potential for e-cigarette condensate to impact primary human osteoblast function, by assessing the secretion of the pro-osteogenic protein Osteoprotegerin (OPG) after low dose condensate exposure. In untreated cells, OPG secretion increased with each timepoint (Figure 2A). Following incubation with either 2nd generation or 3rd generation ECVC for 24 h, OPG secretion declined in a dose dependent manner (Figure 2A,B). Notably, OPG secretion was significantly reduced after exposure to 0.5% ECVC from either 2nd generation (33% ± 1.43% reduction, *p* = 0.015) or 3rd generation (52% ± 0.08% reduction, *p* =< 0.0001) devices (Figure 2A,B), despite this dosage having no effect on osteoblast viability, or morphology (Figure 1). 

**Figure A1:**
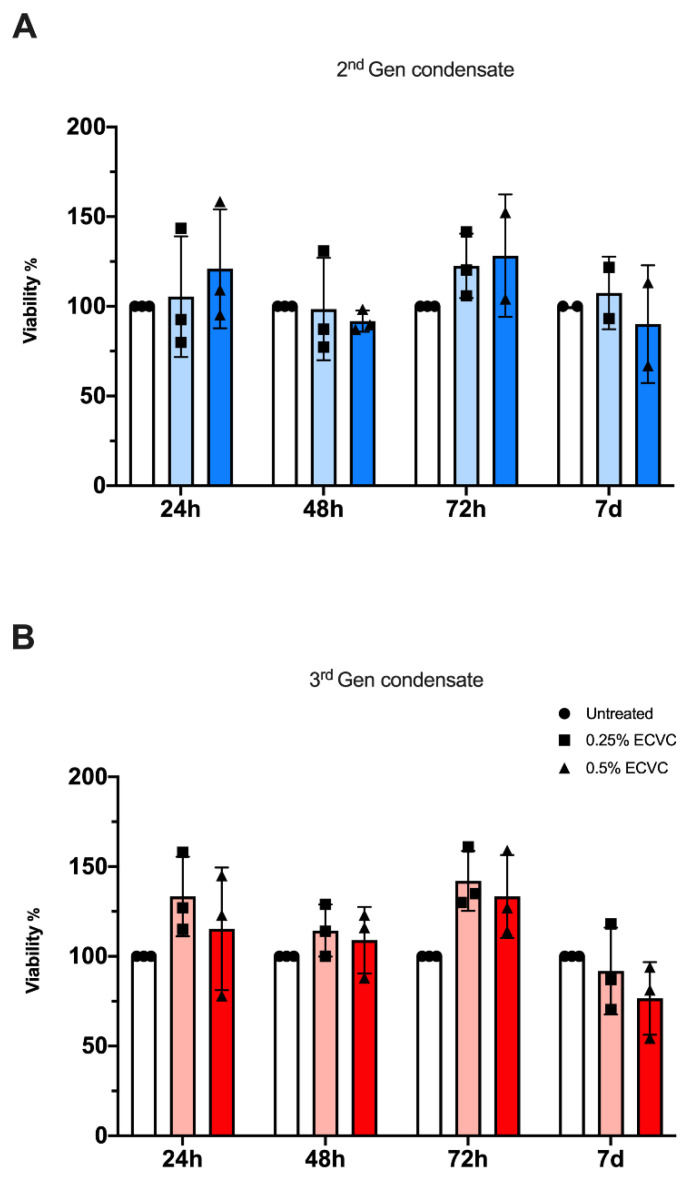
Low dose stimulation of Primary human osteoblasts for up to 7 days does not affect viability. Effect of treating primary human osteoblasts either 2nd (**A**) or 3rd generation (**B**) ECVC at either 0.25% or 0.5% for 24 h-7d on primary human osteoblasts. *n* = 3 patient replicates with 4 biological replicates performed per patient.

Furthermore, over 7 days, OPG secretion continued to increase significantly in untreated osteoblasts in a time dependent manner (Figure 2C). However, continuous exposure to either 0.25% or 0.5% ECVC for 7 days, significantly blunted OPG secretion at each timepoint for both 2nd and 3rd generation devices. By day 7, OPG secretion was approximately 2-fold or 4-fold less than that of untreated control cells for 2nd and 3rd generation devices, respectively, (*p* < 0.0001) (Figure 2C,D), whilst cellular viability and morphology remained unaffected (Figure 2E,F and Figure A1 in Appendix A).

### 3.4. The Antioxidant N-Acetyl Cysteine Rescues the ECVC-Induced Reduction in Osteoblast Viability

**Figure 3:**
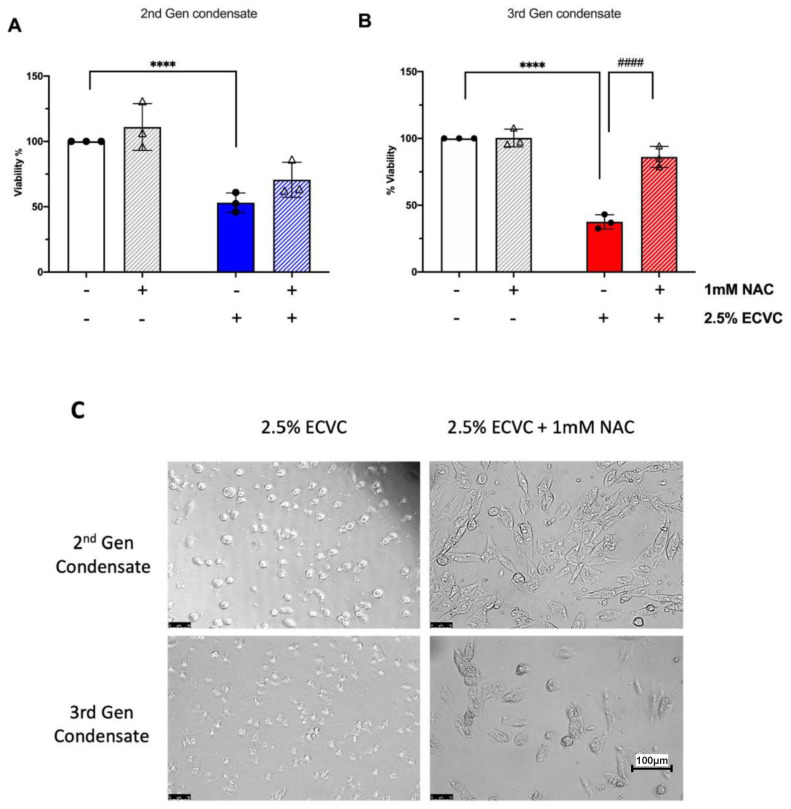
Pharmacological rescue of ECVC induced reductions in human osteoblast viability. (**A**) Effect of 1 mM NAC on human primary osteoblast viability in the absence or presence of 2.5% 2nd generation ECVC for 24 h. (**B**) Effect of 1 mM NAC on human primary osteoblast viability in the absence or presence of 2.5% 3rd generation ECVC for 24 h. (**C**) Representative images of human primary osteoblasts following 24 h exposure to 2nd or 3rd generation ECVC in the presence or absence of 1 mM NAC. Images captured at 20× magnification. Viability was inferred by 4 h incubation with cell titre aqueous assay. *n* = 3 patient replicates, with 3 biological replicates performed per patient. **** *p* < 0.0001 denoting a significant difference to relevant untreated control. #### *p* < 0.05 denoting a significant difference between treatment groups.

To elucidate the mechanism by which ECVC may interact with and affect osteoblast function, we challenged osteoblasts with a toxic dose (2.5%) of ECVC, previously identified to negatively impact osteoblast viability for both 2nd and 3rd generation devices (Figure 1A). These experiments assessed the efficacy of the antioxidant and antialdehyde, NAC to rescue osteoblast viability when given concurrently with condensate challenge. NAC alone had no significant effect on osteoblast viability (Figure 3A,B). However, NAC treatment was able to partially mitigate the toxic effects of ECVC challenge, offering a significant protective effect after the 3rd generation condensate challenge (48.5% restoration, *p* < 0.0001). NAC intervention also mitigated effects on osteoblast morphology following treatment with 2.5% ECVC (Figure 3C).

**Figure 4:**
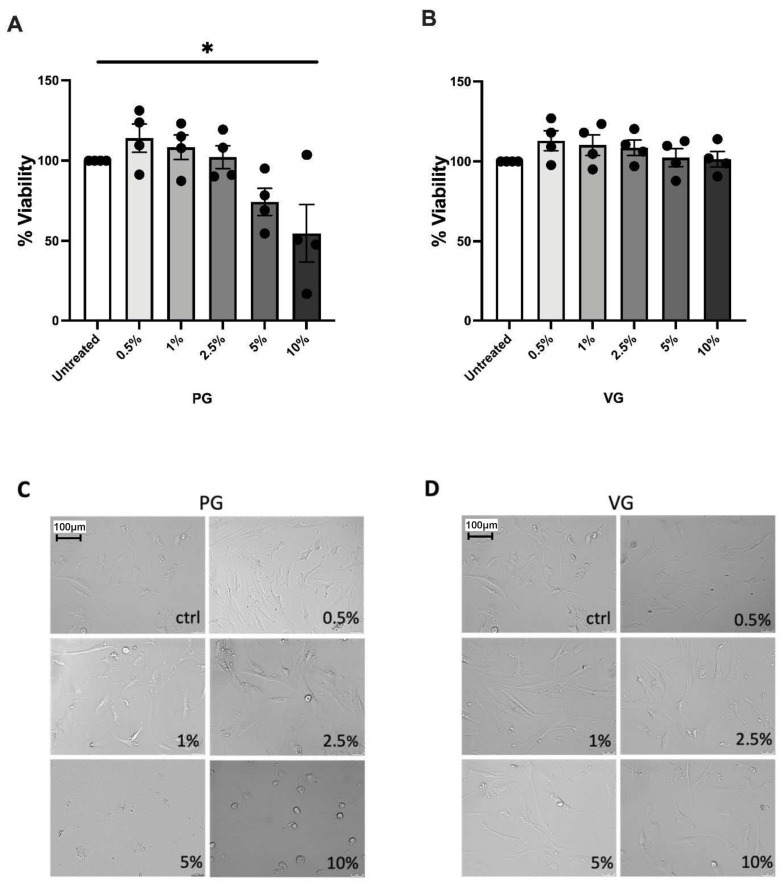
The effect of treatment with e-cigarette constituents on human osteoblast viability. (**A**,**B**) Effect of 24 h treatment of increasing dosages of PG and VG human primary osteoblast viability. (**C**,**D**) Representative images of human primary osteoblasts following 24 h exposure to increasing dosages of PG and VG, images captured at 20× magnification. *n* = 4 Patient replicates with 4 biological replicates performed per patient. * signifies *p* < 0.05 denoting a significant treatment effect by a Kruscal-Wallace test.

Having observed this NAC mediated rescue effect, we next performed a series of experiments in which we treated osteoblasts with the humectants propylene glycol and vegetable glycerine in isolation, to further validate whether e-cigarette vapour components other than nicotine may reduce osteoblast viability (Figure 4). A significant treatment effect of PG on osteoblast viability was observed (*p* < 0.05, Kruskal–Wallace test), with 10% PG decreasing viability up to 80% (Figure 4A) and clearly altered cellular morphology observed with 5–10% dosages (Figure 4C). VG had no significant effect on osteoblast viability or morphology (Figure 4B,D).

## 4. Discussion

This is the first study to demonstrate that e-cigarette condensate exposure reduces human primary osteoblast viability and function in a dose-dependent manner, utilising a model system that accounts for changes in the chemical composition of e-cigarette liquids that occur during vaping.

Treatment of osteoblasts with concentrations of 2.5% ECVC and above, was cytotoxic from both 2nd generation and 3rd generation devices, reducing viability to less than 30% compared to untreated controls. Although there is a lack of data regarding the concentration of nicotine and other vapour constituents delivered to bone following e-cigarette usage, a concentration of 2.5% ECVC (15.5 μM, 3rd generation) as used here is within a physiologically relevant systemic concentration based on reported levels after e-cigarette usage [32]. In concordance with these results, previous studies have demonstrated that cigarette smoke is toxic to human osteoblasts, reducing osteoblast viability in both a concentration and time-dependent manner [33]. More recently, a toxic effect of e-cigarette vapour on a variety of cell types, including alveolar macrophages and epithelial cells has been reported [23,34]. Additionally, Shaito et al. observed both reduced proliferation and impaired osteoblastic differentiation of bone marrow derived mesenchymal stem cells (MSCs) following exposure to e-cigarette aerosol extract [34]. Therefore, e-cigarette use may not only reduce osteoblast viability directly, as observed in this study, but could also reduce MSC-mediated bone repair. Together, these data suggest long-term e-cigarette use could have significant implications for individuals following chronic use and especially individuals with disorders of the skeletal system such as osteoarthritis [35,36], osteoporosis [36] and scoliosis [37], where there is evidence of abnormal bone and/or osteoblast pathology. In addition, this is also likely to impact skeletal remodelling during bone healing following injury, orthopaedic surgery and oral surgery such as dental implants, where exposure to e-cigarette vapour will be in very close proximity to the wounded site. Furthermore, it is also very important to consider the impact of e-cigarettes on adolescent and young adults, who comprise one of the largest cohort of e-cigarette users. Sustained e-cigarette use in such individuals may impair ongoing bone development, leading to reduced bone mineral density into adulthood. Harmful effects of conventional cigarettes and e-Cigarettes have been attributed, at least in part, to nicotine [38]. Our data supports a role for nicotine in driving osteoblast dysfunction, as the impact of nfECVC on osteoblast viability was significantly less than following exposure to ECVC of the same concentration. This effect was particularly apparent for second-generation device condensate. However, it should be noted that although osteoblast viability was greater following 10% 2nd gen nfECVC exposure in comparison to the 3rd generation device, cellular morphology appeared abnormal. This could be explained due to the viability assay fundamentally being based on cellular metabolism, therefore it is possible that although stressed and so losing typical morphology, the cells treated with the 2nd generation condensate were still more metabolically active. Whereas those treated with the 3rd gen condensate, quickly began to die after treatment. The considerable sustained impact of 3rd generation nfECVC on osteoblast viability may be attributable to differences in vapour constituent content. In addition to nicotine, e-Cigarette vapour also contains carrier agents/humectants including propylene glycol and vegetable glycerine. Thermal degradation of these carrier compounds generates reactive carbonyl species at similar concentrations to those seen in cigarettesmoke (~5 μg·puff^−1^) [39] and in some cases, in excess of cigarette smoke (200 ug·puff^−1^) [21]. Importantly, reactive carbonyl species have been demonstrated to reduce proliferation, increase cell death and inhibit both osteoblast alkaline phosphatase activity and mineralisation [40,41,42]. Additionally, e-cigarette liquids also generate a considerable amount of short-lived, highly reactive free radicals (>10^13^ molecules/puff) [21,43,44,45] upon vapourising. ROS are reported to induce apoptosis of osteoblasts, as well as inhibit osteoblastic differentiation, reducing osteoblast number and impairing function [45,46,47]. Furthermore, Bai et al. present evidence that increased intracellular ROS can stimulate the expression of RANKL in human osteoblast-like cells, which would be expected to promote osteoclast activity and bone resorption [48]. Collectively, these findings suggest that ROS not only reduces osteoblast mediated bone formation, but may also increase bone resorption through activation of osteoclasts, ultimately resulting in reduced bone density. Critically, increasing battery size and decreased coil resistance result in increased amounts of both ROS and RCS being generated per puff by newer generation e-cigarette devices, such as the 3rd generation device used in this study [20,22,45]. Unlike previous studies that treat cells with e-cigarette liquids directly from the bottle, our model system accounts for changes in chemical composition that occur upon vaping. Therefore, it is possible that the nicotine free ECVC from the 3rd generation device used in this study contained a greater amount of reactive species compared to 2nd generation devices, in turn mediating a greater impact on osteoblasts. To investigate this possibility, we treated osteoblasts with ECVC in the presence of NAC, an antioxidant that has been reported to protect against ROS and reactive aldehydes [49]. Following treatment with 3rd generation ECVC, NAC provided a significant protective effect, restoring viability to the level of control osteoblasts. This suggests that the toxic effects of ECVC may indeed be mediated in part by increased levels of reactive species. We also demonstrate that components of e-cigarette condensate other than nicotine, including the humectant PG, also significantly impaired osteoblast viability and altered cellular morphology. Emerging data in gingival and airway epithelial cells has also shown cytotoxic, inflammatory and metabolic effects of such compounds, widely regarded as inert carrier agents [50,51]. It should be noted that repeated exposure to vapour generated from humectants alone over a 6 month period had no significant effect on bone morphology in mice [52]. However, the potential impact of such humectant exposure on osteoblast/osteoclast function and bone turnover remains to be determined and therefore potential implications to processes such as bone healing still need to be considered. Indeed, although we observed no reduction in viability following VG treatment, recent work has demonstrated that VG exposure can impact chloride channel expression [53]. Therefore, non-toxic doses of VG and PG may still have considerable effects on osteoblast function, similarly to the effect non-toxic doses of ECVC had on OPG secretion as we report in this study. Collectively these data emphasise that further studies are necessary to understand the effect of chronic exposure to humectants and other components of e-cigarettes on human bone, especially following chronic use. Additionally the need for such studies is paramount, as e-cigarette devices are continually innovating, leading to ever greater power output and therefore increasing burden of RCS per puff and so in turn potential harm is only likely to increase 

In line with the detrimental effect of ECVC on osteoblast viability, we also found that ECVC impaired the functional ability of osteoblasts by reducing their secretion of OPG, the decoy receptor for RANK ligand and a key regulator of bone turnover. Previous studies have examined the impact of conventional cigarette smoking on serum OPG levels, concluding that levels were significantly reduced in smokers [54]. In addition, OPG:RANKL is significantly reduced in patients who smoke, suggesting smoking may drive bone resorption [54]. Here, we also observed that OPG production by osteoblasts was significantly reduced following treatment with ECVC at 24 h, suggesting that the effects of e-Cigarette use and conventional cigarette smoking on osteoblast function maybe comparable. We did attempt to measure RANKL protein content in osteoblast supernatants by ELISA, however protein concentrations were below the lower limit of detection of our assay. Critically, as e-cigarette use is typically chronic, we also found that stimulation using concentrations of ECVC from either 2nd or 3rd generation devices, that did not impair osteoblast viability (0.25% and 0.50%) was sufficient to elicit a sustained reduction in OPG secretion for up to 7 days. This suggests long term use of e-cigarettes could lead to chronic suppression of OPG, promoting greater bone resorption Such non-toxic effects could also extend to suppression of other critical cellular functions of osteoblasts, such as alkaline phosphatase activity, or expression of genes such as COL1A1, in turn reducing extracellular matrix secretion. This carries clear clinical implications for a number cohorts including; adolescent users still undergoing bone development, in addition to individuals with bone associated disease such as osteoporosis, and those recovering from injury and surgery as discussed above. In line with this, it may also be important to consider chronic e-cigarette use in the context of DNA damage and ageing. Although e-cigarettes are widely thought to produce fewer carcinogenic compounds relative to cigarettes, nicotine nitrosation does indeed occur following e-cigarettes use, inducing the formation of the DNA adducts O(6)-methyl-deoxyguanosines and cyclic γ-hydroxy-1, *N2*–propano-dG [55]. Additionally, the presence of DNA adducts associated with reactive carbonyl species, such as acrolein, have also been reported following e-cigarette use in humans [56,57,58]. Therefore, it seems reasonable to assume that chronic systemic delivery of such compounds following e-cigarette use could reduce DNA repair in bone, in turn driving an accelerated bone ageing phenotype as described by Chen et al. [59]. Consequently, this may compound dysfunctional bone remodelling and further contribute to reduced bone mass and increased fracture risk, particularly in older users. 

Due to the difficulty in replicating e-cigarette use in vitro, this study does have limitations. Firstly, although we have used a range of does in this study, concentrations of nicotine and other metabolites in the vapour condensate may not represent localised interstitial concentrations. Smoker and vaper plasma nicotine will of course vary greatly dependent on personal addiction level. However, ex-smokers will vape to meet their individual nicotine addiction needs [18] and as such, it is likely tissue exposure will remain comparable between these groups. Smoker urinary nicotine and nicotine metabolites (assessing chronic exposure) have been quantified in a range from 7–338 μM [60]. Here, we have delivered non-toxic nicotine doses of 0.82 μM (0.5% 2nd generation challenge); and 3.1 μM (0.5% 3rd generation challenge) [32]. Whilst lacking definitive data for comparison, these low dose challenges are well within a feasible physiological range [32]. Secondly, osteoblasts were only stimulated with one dose of ECVC, and therefore we can only speculate that repeated daily usage of chronic e-cigarette use would have a similarly detrimental effect on osteoblast viability. 

## 5. Conclusions

In summary, we have demonstrated that ECVC has a negative effect on both osteoblast viability and function, with these effects being mediated, in part, by nicotine-dependent mechanisms and also reactive carbonyl species derived from e-liquid humectants as summarised in Table 1. Reduced osteoblast viability, coupled with a reduction in OPG secretion as observed following ECVC treatment, may lead to increased bone resorption following chronic exposure, in turn potentially impacting bone development in younger users, while increasing bone associated disease progression and negatively impacting orthopaedic and dental surgeryoutcomes.

## Figures and Tables

**Table 1:** A summary of key findings.

	2nd Generation Device		3rd Generation Device		Humectants	
	ECVC	nfECVC	ECVC	nfECVC	PG	VG
Dose to significantly reduce osteoblast viability (24 h)	2.5%	10%	1%	2.5%	Significant treatment effect 0.5–10%	No significant effect
Dose to significantly reduce osteoblast OPG secretion (24 h)	0.5%	-	0.5%	-	-	-

## Data Availability

Not applicable.
